# Clinical Applications of Artificial Intelligence in Glaucoma

**DOI:** 10.18502/jovr.v18i1.12730

**Published:** 2023-02-21

**Authors:** Siamak Yousefi

**Affiliations:** ^1^Department of Ophthalmology, University of Tennessee Health Science Center, Memphis, TN, USA; ^2^Department of Genetics, Genomics, and Informatics, University of Tennessee Health Science Center, Memphis, TN, USA

**Keywords:** Artificial Intelligence, Convolutional Neural Network (CNN), Deep Learning, Glaucoma, Machine Learning, Ophthalmology

## Abstract

Ophthalmology is one of the major imaging-intensive fields of medicine and thus has potential for extensive applications of artificial intelligence (AI) to advance diagnosis, drug efficacy, and other treatment-related aspects of ocular disease. AI has made impressive progress in ophthalmology within the past few years and two autonomous AI-enabled systems have received US regulatory approvals for autonomously screening for mid-level or advanced diabetic retinopathy and macular edema. While no autonomous AI-enabled system for glaucoma screening has yet received US regulatory approval, numerous assistive AI-enabled software tools are already employed in commercialized instruments for quantifying retinal images and visual fields to augment glaucoma research and clinical practice. In this literature review (non-systematic), we provide an overview of AI applications in glaucoma, and highlight some limitations and considerations for AI integration and adoption into clinical practice.

##  INTRODUCTION

Artificial Intelligence (AI) applications in ophthalmology have shown significant advancements due mainly to the availability of computational platforms, generation of large annotated ocular images, and emergence of AI algorithms. Several landmark studies have highlighted the effectiveness of AI applications in screening, referral, and diagnosis of different ocular conditions.^[1,2,3]^

AI is a broad term encompassing a wide range of subfields including image processing and expert systems, in which models are preprogrammed and thus require domain knowledge (i.e., human expertise to guide the programmer). In contrast, another subfield of AI, machine learning, can learn from data and identify the outcome of new circumstances without being explicitly programmed. Machine learning models can be further sub-divided to include *supervised learning *in which the label of data is available and *unsupervised learning* in which the labels of the data are unknown. *Deep convolutional neural networks* (CNNs) are supervised machine learning models that utilize a stack of hidden layers composed of artificial neurons to emulate human brain in the learning and recognizing process.

**Figure 1:**
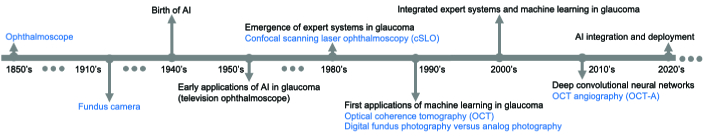
Timeline of major retinal imaging instruments and landmark artificial intelligence applications in glaucoma. Introduction of landmark imaging instruments are listed in blue and AI events are provided in black. AI, artificial intelligence; OCT, optical coherence tomography; OCTA, OCT angiography.

Figure 1 shows a broad timeline of major retinal imaging instruments and AI applications in glaucoma. The ophthalmoscope was invented by Helmholtz in the 1850s, which revolutionized ophthalmology, as it allowed direct visualization of the retina and optic disc. Introduction of fundus photography in the 1910s allowed documentation of the status of the retina, thus enhancing the monitoring and management of glaucoma patients. AI was born in the 1940s and some researchers attempted to apply some AI techniques, including classical image processing, to locate the optic disc in retinal frames generated from a television ophthalmoscope in 1950s. However, it was not until the 1980s that some expert systems were applied to retinal images to quantify optic disc properties useful in detecting glaucoma. Subsequently, AI models have been broadly applied to different aspects of glaucoma including retinal and optic nerve image and visual field (VF) quantification, screening, referral, diagnosis, forecasting (prediction), prognosis, and monitoring.

Early machine learning models in glaucoma were based on neural networks and attempted to diagnose glaucoma from VFs in the 1990s.^[4,5]^ Thereafter, various machine learning models were applied to diagnose glaucoma based on fundus photographs, optical coherence tomography (OCT), OCT angiography, and other ocular and demographic parameters, followed by various deep learning models in the 2010s.^[6]^–^[31]^

##  METHODS

In this review, we used search combinations of “artificial intelligence”, “machine learning”, “neural networks”, “deep learning”, “glaucoma screening”, “glaucoma diagnosis”, “glaucoma progression”, “segmentation”, and “image annotation” in Google and PubMed to review broad applications of AI in glaucoma. We categorized AI applications in glaucoma into four major groups: (1) applications in retinal imaging and VF quantification; (2) applications in screening, referral, diagnosis, and forecasting (prediction); (3) applications in monitoring and progression detection; and (4) applications in estimating functional parameters from structural factors. We then highlighted some of the limitations and challenges of integrating these AI models into clinical care.

### AI in Glaucoma Image and Data Quantification, and Characterization

Retinal imaging and VF testing in conjunction with clinical examinations form the primary basis for assessment and diagnosis of glaucoma.^[32]^ While *color fundus photography* has long been used to document retinal status, recent *OCT imaging* provided three-dimensional views of retinal layers and optic nerve head structures.^[33]^ In addition to these modalities, functional assessment typically performed via *standard automated perimetry (SAP)* ^[34]^ has remained a standard practice for diagnosis and prognosis of visual function in patients with glaucoma.^[34,35]^ These three imaging modalities comprise the major components of glaucoma assessment. As such, improvements in the quantification and characterization of retinal images and VFs could promote objectivity, improve consistency in glaucoma assessment, and set a common ground for research and clinical practice. For instance, interpreting vertical cup-to-disc ratio (CDR) may facilitate glaucoma diagnosis^[36]^ as CDR is a major risk factor; likewise, monitoring RNFL thickness may facilitate prognosis as thinning of RNFL is a hallmark of glaucoma progression.^[37]^ Quantification of VFs in the form of glaucoma-induced patterns of VF loss could also facilitate diagnosis and assist therapy adjustment and prognosis plan optimization based on the shape, type, and depth of defect with consideration of the patient's quality of the life.^[38]^

**Figure 2:**
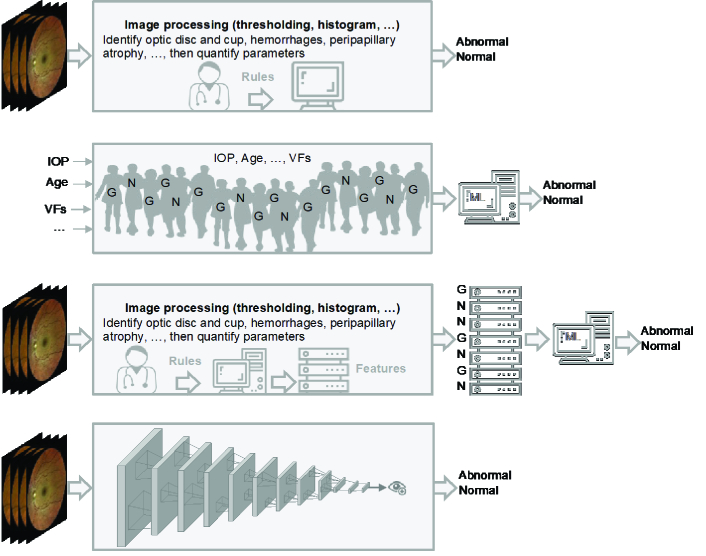
Evolution of AI in glaucoma. **First row**: Image processing and expert systems were used to identify glaucoma landmarks or features (such as cup-to-disc ratio or hemorrhages) from retinal images with the assistance of a glaucoma specialist and glaucoma landmarks are identified. **Second row**: Numerical parameters like raw visual fields (VFs), intraocular pressure (IOP), and age from normal and glaucomatous subjects (presented as *N* and *G*) are input to a conventional machine learning model (e.g., neural network) without glaucoma specialist assistance and diagnosis is made. **Third** **row**: Image processing and expert systems were used to quantify glaucoma landmarks (extract features) with the assistance of a glaucoma specialist then quantified parameters (features) from normal and glaucomatous subjects are fed to a conventional machine learning model to make diagnosis. **Fourth row**: Retinal image is fed to an end-to-end deep learning model and the diagnosis is made without assistance from a glaucoma specialist.

AI models have been proposed to quantify retinal images as early as the 1950s [Figure 1 & Figure 2: top row]. In conventional AI models (image processing and expert systems), the role of human expertise in hand-crafting algorithms to quantify glaucoma-induced changes and lesions from retinal images was critical. For instance, optic disc and cup boundaries were automatically detected based on various classical image processing techniques that typically require human expertise in the process. Retinal fundus image processing usually requires pre-processing steps to prepare and enhance the image for feature extraction (identifying landmarks). Many of the image processing techniques include histograms equalization and morphological (shape) filtering, and active contours. More involved processes such as gradient vector flow was used to delineate optic disc and cup boundaries in early AI models.^[6]^ As CDR is a major glaucoma risk factor, many of the follow-up AI models focused on localization and quantification of the optic disc and cup in fundus photographs to identify CDR. Hoover et al localized and quantified optic disc and cup based on the information derived from blood vessels.^[39]^ Chrastek et al suggested an automated model for ONH segmentation and quantification based on morphological operations, Hough transform, and active contours.^[11]^ Wong et al used several classical image processing steps to segment the optic cup and disc from retinal images then used a fusion network to combine quantified parameters and subsequently employed an SVM classifier to discriminate normal eyes from glaucomatous eyes.^[40]^ Follow-up studies on fundus photographs also have applied broad classical image processing techniques including edge detection, morphological filtering, adaptive deformable filters, and active contours to quantify optic disc characteristics to assist glaucoma diagnosis [Figure 2: third row).41-45

Emerging deep CNN models however changed the paradigm from manual feature engineering to automatic end-to-end quantification of color fundus images (Fig. 2: fourth row]. One of the first applications of deep learning in quantifying optic disc and cup from fundus photographs was introduced in 2015.^[12]^ They developed a deep learning model using two publicly available datasets of fundus images and segmented optic disc and cup and computed the degree of vessel kinking integrated with prior knowledge about retinal structures to quantify fundus images. Other models have obtained AUCs up to 0.92 in detecting glaucoma from the quantified retina and ONH characteristics based on fundus images.^[46]^

Fundus photographs were traditionally used to document retinal structure. However, with the introduction of OCT^[47]^ in the 1990s, this modality soon became popular and is now an indispensable component of glaucoma assessment.^[33]^ OCT quantification is thus highly rewarding yet challenging because OCT provides a significantly lower resolution compared to color fundus photographs, and lesions and characteristics are not typically as obvious as those in fundus photographs. Moreover, the shadows generated due to blood vessels pose additional quantification challenges.^[33,48]^ Nevertheless, OCT provides substantial retinal structural information in three dimensions and its quantification can be highly useful. For these reasons, OCT quantification and interpretation has always been an active area of research since its invention. Like color fundus photographs, the conventional AI methods to quantify OCT images typically include classical image preprocessing techniques such as linear or non-linear filtering, edge detection, and local texture analysis. Koozekanani et al developed an algorithm for OCT retinal layer segmentation based on classical edge detection and Markov modeling. Based on 1450 OCT B-scans, the derived retinal thickness measurements deviated from the ground truth thicknessed by less than 10 microns for ∼ 74% of the B-scans and by less than 25 micron for ∼ 99% of the B-scans.^[49]^ Ishikawa et al proposed an algorithm based on adaptive thresholding technique to segment macular OCT images and subsequently used quantified parameters to diagnose glaucoma. Based on a dataset with about 60 OCT images, they obtained AUCs up to 0.97 for discriminating normal eyes from eyes with established glaucoma.^[50]^ Follow-up models further improved the segmentation accuracy. For example, Kafieh et al developed an OCT segmentation model based on local image textures and diffusion mapping to quantify retinal layers. They evaluated their model using 23 OCT images collected from normal and glaucomatous eyes and obtained retinal layer quantifications with lower than ∼ 8 microns of thickness error.^[51]^

Emerging deep CNN models however have transformed OCT image quantification from manual feature extraction and annotation to automatic end-to-end quantification. Recent deep learning models provide detailed quantifications of OCT layers as well as information regarding existing pathologies and underlying ocular condition.^[13]^–^[17,52,53]^

**Figure 3:**
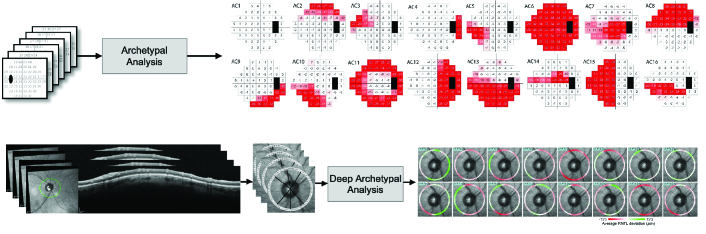
Visual field (VF) and optical coherence tomography (OCT) image quantification. Top: VFs were quantified to 18 prominent patterns of VF loss based on classical archetypal analysis. Bottom: OCT circle scans were quantified to 16 patterns of RNFL loss based on deep archetypal analysis.

In terms of VFs, various methods have been proposed to summarize, quantify, and annotate VFs. Garway-Heath et al^[54]^ developed a model to map VF test locations on optic nerve structure to better quantify the relationship between localized VF and retinal nerve fiber layer (RNFL) loss. Some groups focused on identifying local patterns of glaucomatous VF loss then classifying and quantifying the severity levels based on subjective assessments.^[55,56]^ However, manual identification and classification of VF patterns is labor-intensive and requires high levels of expertise that may be prone to inter-and intra-reader variability.^[57,58]^ Subsequently, numerous automated models based on conventional machine learning approaches were proposed to identify and classify patterns of VF defect using unsupervised Gaussian mixture modeling (GMM), archetypal analysis, or deep archetypal analysis.^[22]^–^[31]^ Most of the AI models for quantifying and annotating VFs are based on conventional unsupervised learning. Figure 3 shows how classical archetypal analysis applied can be used to decompose VFs into 18 prevalent patterns of VF loss [Figure 3: top panel] and decomposition of OCT circle scans to 16 prevalent patterns of RNFL loss [Figure 3: bottom panel]. Such a model can decompose VF or OCT data to a weighted combination of these prevalent patterns and even be used for subsequent detection of glaucoma progression.^[22]^–^[31]^

In terms of clinical applications, most of the commercially available OCT imaging instruments provide some level of OCT image quantification, interpretation, and visualization. The widely used Humphrey VF analyzer provides several summary parameters including mean deviation (MD), pattern standard deviation (PSD), and visual field index (VFI), and regional parameters such as glaucoma hemifield test (GHT). However, this is not the case for most (if not all) fundus cameras. But, as OCT is predominantly used in glaucoma clinical care, color fundus photograph quantification may not be a major limitation in clinical applications. Nevertheless, fundus cameras are usually cheaper and more portable than current commercialized OCT instruments, and thus may be more appropriate for community-based glaucoma screening. Thus, while innovative AI models may be applied to OCT and VF data to provide more objective and consistent parameters in glaucoma clinical practice, AI may augment color fundus photography in quantifying more specific and sensitive parameters to enhance community-based and glaucoma screening programs.

#### Clinical considerations

The effectiveness of using optic nerve characteristics in detecting glaucoma has been investigated extensively. Damms et al observed that vertical CDR best suits glaucoma screening, whereas the rim area is more appropriate for detecting progression.^[59]^ A follow-up study, however, found that localized rim area led to the highest specificity of 90% and sensitivity of 91% for discriminating glaucoma from normal eyes based on computerized raster tomography.^[60]^ A recent study suggested vertical CDR as the most important feature for diagnosing glaucoma based on color fundus photographs.^[36]^ Therefore, more accurate quantification of parameters such as CDR and RNFL thickness profiles may augment clinical care and improve more objective glaucoma assessment and diagnosis. Moreover, tracking the quantified parameters over time may facilitate a more objective and accurate monitoring and progression detection.

### Applications of AI in Glaucoma Screening, Referral, Diagnosis, and Forecasting

While the first applications of image processing in glaucoma dates back to the1950s, the first applications of machine learning models in glaucoma dates to the 1990s when several teams applied neural networks to VFs for glaucoma diagnose [Figure 2: second row].^[4,5]^ Numerous follow-up neural network-based models were proposed to diagnose glaucoma based on VFs.^[61]^–^[64]^ As VFs were composed of numerical values of threshold sensitivity or total deviations, they provided the optimal input to neural networks which may explain the extensive utility of early neural network models for glaucoma diagnosis based on VFs. A study conducted by Chan and colleagues compared several machine learning models including multilayer perceptron (MLP), support vector machine (SVM), linear and quadratic discriminant analysis, mixture of Gaussian (MOG), and mixture of generalized Gaussian (MGG) in diagnosing glaucoma based on VFs and found that machine-learning-type classifiers provided higher accuracy compared to best VF indexes from the STATPAC software in diagnosing glaucoma.^[7]^ Other teams utilized various machine learning classifiers such as SVM, discriminant analysis, bagging, and ensemble learning to identify glaucoma based on VFs.^[8]^–^[10]^

Raw VFs provide a small grid of numbers (typically fewer than 9*9), thus, in contrast to color fundus and OCT images, VFs are basically inappropriate for deep CNN analysis. As such, some researchers have applied deep CNN models on VF printouts (reports) rather than raw VF numbers. Li et al developed a deep CNN model based on over 4000 VF printouts and obtained an AUC up to about 0.87 in differentiating normal from glaucomatous VF while an SVM model achieved an AUC of 0.67 and glaucoma experts achieved an AUC up to 0.62.^[65]^ A recent deep CNN models utilized over 16,000 VFs and obtained AUCs up to 0.93 for diagnosing glaucoma.^[66]^

Glaucoma is characterized by progressive structural loss of retinal ganglion cells (RGCs), therefore structural evaluation is a critical step in glaucoma assessment. Some early studies showed that RVM and SVM classifiers can discriminate glaucoma from normal eyes using RNFL thickness measurements derived from scanning laser polarimetry (SLP) instruments with AUROCs of up to 0.94.^[67]^ Others generated ONH parameters (after a manual outlining of the optic disk border) such as cup's volume, depth, and shape as well as rim's characteristics from CSLO instruments to diagnose glaucoma.^[68]^ While AI models were applied to SLP or cSLO-derived ONH and RNFL parameters (generated by the instruments), diagnostic AI models based on color fundus images were more involved as instruments typically did not provide quantified parameters. As a result, the AI models were required to first quantify characteristics (extract features) then learn those quantified features for glaucoma diagnosis. Bock et al developed image processing models (conventional AI) for glaucoma screening that first preprocessed color fundus photographs and generated different generic features and then performed dimension reduction to lower the number of features. These features were then combined, and a glaucoma risk index was generated, which achieved an AUC of 0.88 in screening glaucoma.^[69]^ Other AI models used a combination of texture and higher order spectra features from color fundus photographs, then employed numerous machine learning models including SVM, naive Bayesian, and random-forest and obtained an accuracy up to 91% based on the random-forest classifier.^[70]^ Cheng et al first segmented the optic disc and cup using histograms of pixel intensities, neighbor statistics, and incorporation of location pixel information to first compute CDR for glaucoma screening and thus obtained AUCs up to 0.82 based on two independent datasets.^[71]^ More complex learning processes such as multi-task learning has been used to detect glaucoma and several other ocular conditions from fundus photographs.^[72]^

Deep learning models could, however, learn complex glaucoma features using several layers of neurons in an end-to-end process. One of the first deep CNN models in glaucoma used a network with four convolutional layers and two fully connected layers. This model obtained AUCs up to 0.88 for detecting glaucoma from fundus photographs based on two different publicly available datasets.^[73]^ Some of the recent deep CNN models applied to fundus photographs have reached AUROCs up to 0.99 for glaucoma diagnosis.^[74,75,76,78]^ Other deep learning models have obtained AUC up to about 0.97 for glaucoma screening and AUC up to 0.94 for glaucoma referral.^[36,79]^ A recent meta-analysis paper analyzed the accuracy of seventeen deep learning-based studies that utilized 30 different patient cohorts and reported an AUC of 0.93 (95% CI 0.92–0.94) for diagnosing glaucoma based on color fundus photographs.^[80]^

As OCT has become a dominant imaging modality for glaucoma assessment,^[33]^ numerous teams have explored the utility of glaucoma diagnosis based on OCT. Some studies have shown the usefulness of the OCT-derived RNFL parameters in distinguishing normal eyes from eyes with glaucoma without the utilization of AI models. Based on 94 normal subjects and patients with early glaucoma, Bowd et al obtained an AUC of 0.91 using COT parameters and showed the accuracy based on OCT was superior to scanning laser polarimetry (SLP), frequency-doubling technology (FDT), and short-wavelength automated perimetry (SWAP) in discriminating normal eyes from eyes with early glaucoma.^[81]^ Another study used RNFL thickness parameters of 95 age-matched normal and glaucomatous eyes and obtained accuracies up to about 90% in distinguishing normal eyes from glaucomatous eyes based on the commercially available OCT instruments of Stratus and Cirrus (Carl Zeiss Meditec, Dublin, CA).^[82]^ These results were promising, and several follow-up studies showed that OCT can discriminate normal eyes from glaucomatous eyes with AUCs ranging from 0.89 to 0.96 based on RNFL or GCIPL thickness parameters derived from macula or ONH OCT images.^[37,83,84,85,86,87]^ These studies showed the capability of OCT-derived retinal parameters in diagnosing glaucoma without employing machine learning models.

The capability of machine learning models in improving the diagnostic accuracy for glaucoma based on OCT-derived retinal parameters were investigated further. Burgansky-Eliash et al evaluated five conventional machine learning classifiers including linear discriminant analysis, SVM, recursive partitioning and regression tree, generalized linear model, and generalized additive model based on OCT-derived parameters of 89 normal and glaucomatous eyes and obtained the best AUC of 0.98 (specificity of 95% and sensitivity of 92.5%) for discriminating normal eyes from glaucomatous eyes using an SVM classifier.^[88]^ Follow-up AI models based on OCT-derived RNFL thickness measurements collected from 152 normal and glaucomatous eyes using SVM and ANN machine learning classifiers obtained AUCs up to about 0.99^[89]^ and another neural network-based model evaluated RNFL-derived OCT segmentation strategies and obtained an AUC up to 0.85 for glaucoma diagnosis.^[90]^

Recent deep CNN models have also been applied to OCT-derived retinal parameters to diagnose glaucoma. Asaoka et al applied a deep learning model on over 4000 grids (8*8) of macular OCT-derived RNFL and ganglion complex layer (GCL) thickness profiles and obtained an AUC about 0.94.^[18]^ Follow-up deep learning models have obtained AUCs up to 0.99 based on OCT-derived retinal parameters.^[19,20,21]^ Deep learning approaches have also been applied to raw un-segmented OCT images for glaucoma diagnosis. Ran et al developed a multi-task three-dimensional (3D) deep learning model to diagnose glaucoma based on over 8000 raw volumetric OCT scans collected from multiple institutes and obtained AUCs in the range of 0.86 to 0.90.^[91]^ A recent meta-analysis investigated five different deep learning studies that analyzed six cohorts of OCT and reported an AUC of about 0.96 (95% CI 0.94–0.99) for diagnosing glaucoma based on deep learning models. When averaged across the cohorts, the pooled sensitivity was 0.94 (95% CI 0.92–0.96) and pooled specificity was 0.95 (95% CI 0.91–0.97).^[80]^

Machine learning models have been applied to other retinal imaging modalities as well. OCT angiography (OCTA), a recent imaging technology in ophthalmology, provides high-resolution images of retinal vasculature structure and function that are appropriate for deep learning models.^[92]^ OCTA-derived vasculature parameters have shown great promise in discriminating normal eyes from glaucomatous eyes without using any AI model.^[93,94,95]^ As OCAT is a newer technology, a limited number of AI models have explored this modality to date. A recent study investigated the capability of deep learning and conventional machine learning classifiers to diagnose glaucoma based on 405 OCTA images and quantified parameters. The best AUC of the gradient boosting classifier (GBC) model based on quantified OCTA parameters was 0.89 while a deep learning model based on a VGG16 architecture achieved an AUC of 0.93 based on radial peripapillary capillary en face OCTA images of the ONH.

Most applications of AI models have been centered around glaucoma detection for screening and diagnosis purposes, while forecasting glaucoma could play an important role in identifying those with future disease development and potential vision loss. Thakur et al developed a deep learning model based on over 60,000 fundus photographs to forecast glaucoma before disease development. They achieved AUCs up to approximately 0.77 and 0.88 for forecasting glaucoma four to seven years and one to three years before onset, respectively. Their model achieved an AUC of about 0.95 once tested to diagnose glaucoma.^[77]^ Other forecasting models are usually centered around predicting future VF or OCT parameters. Wen et al developed a model to forecast future VF tests (up to 5.5 years) from current VF tests using deep learning based on more than 30,000 VFs and obtained average point-wise mean absolute errors (MAE) of about 2.5 dB.^[96]^ Sedai and colleagues developed a deep learning model to forecast RNFL thickness measurements from raw OCT and quantified RNFL thickness measurements, VFs, and clinical data collected from multiple visits, and reached mean MAEs as low as about 1.8 micron in estimating global RNFL thickness across normal eyes, and eyes with suspect and established glaucoma.^[97]^ Such validated models may facilitate personalized patient care by determining the most appropriate inter-visit schedule for timely interventions.

#### Clinical considerations

While two autonomous AI-enabled models have received US FDA approvals for screening diabetic retinopathy and macular edema,^[98,99]^ no autonomous AI models have yet received FDA approval in glaucoma screening, diagnosis, or prognosis. It should be noted that most of the commercially available VF and OCT instruments already include some AI-enabled quantification and interpretation tools, and the evidence summarized above, strongly suggests that autonomous AI models would be warranted for glaucoma screening, diagnosis, and forecasting. Assistive AI models may benefit glaucoma clinical practice and augment clinical assessment while autonomous AI models may provide greater benefit to population-based screening. Nevertheless, various hurdles remain for full development and integration of assistive and autonomous AI models in glaucoma, as discussed in Section 6.

### Applications of AI in Glaucoma Prognosis and Monitoring

Detecting glaucoma-induced structural and functional loss is critical for preserving vision and maintaining quality of life of patients with glaucoma. However, identifying glaucoma-induced vision changes by inspecting a sequence of fundus photographs, OCT images, and VFs can be perplexing at both ends of the glaucoma spectrum - in the early stages of the disease, where structural and functional deficits are subtle; or in the late stage of the disease, where OCT is unable to provide required dynamic ranges (flooring effect) and VF presents significant VF variability.^[100,101]^ Early methods for detecting glaucoma progression introduced in the 1990s include: Advanced Glaucoma Intervention Study (AGIS) criteria;^[102]^ Collaborative Initial Glaucoma Treatment Study (CIGTS) criteria,^[103]^ and the widely used Guided Progression Analysis (GPA).^[35]^ All these models provided event-based approaches utilizing ad-hoc rules to detect VF progression.

Point-wise linear regression (PLR)^[104]^ and Permutation of PLR (PoPLR)^[105]^ have provided trend-based approaches utilizing linear regression to identify VF progression. Other follow-up models used statistical analysis of summary or regional parameters such as VFI or mean deviation (MD) to detect glaucoma progression based on VFs.^[106,107,108]^ More complex models based on structure–function relationship using dynamic estimates of the current glaucoma state and velocity of progression over time showed improved accuracy over the ordinary linear regression approaches.^[108]^ These methods have used mathematical and statical approaches to detect progression.

Lin et al introduced one of the first applications of machine learning models in detecting glaucomatous progression based on VFs.^[109]^ They obtained an AUC of 0.92 (average specificity and sensitivity of 88% and 86%, respectively) using a neural network with three hidden layers. Sample et al introduced one of the first unsupervised machine learning-based models to detect glaucoma progression.^[110]^ They developed an unsupervised variational Bayesian model and identified several prominent patterns of VF loss. They identified the progression of glaucoma across these patterns. Most of the follow-up machine learning models also used unsupervised machine learning models to analyze VFs.^[25,28,111,112]^ As VF testing was an older technology compared to OCT, most of the early glaucoma progression models have been applied to VFs.

Wollstein et al investigated the utility of OCT-derived RNFL thickness measurements in detecting glaucoma progression and reported that OCT was more sensitive than VF in detecting glaucomatous progression.^[113]^ Similarly, other studies also showed the utility of OCT in detecting glaucoma progression.^[114]^ Yousefi et al evaluated the usefulness of several supervised machine learning models to detect glaucoma based on VFs and OCT parameters and reported the superiority of OCT in detecting glaucoma progression compared with VFs.^[115]^ While unsupervised archetypal analysis has been used for detecting glaucoma progression,^[116]^ a recent study used deep archetypal analysis to identify patterns of VF loss and then used some of those patterns for detecting ocular hypertensive patients with future rapid glaucoma progression (rate of MD loss faster than –1 dB/year).^[117]^ The application of deep CNN models in detecting glaucoma progression, however, has been limited. A recent study showed the effectiveness of a convolutional long short-term memory (LSTM) neural network that was trained and tested on over 670,000 VFs in identifying glaucoma progression from VFs with AUCs from 0.79 to 0.82.^[118]^

#### Clinical considerations

Most of the early applications of AI in detecting glaucoma progression have been focused on VFs. This may be explained by two facts. First, the longer existence of VF testing technology in glaucoma care has resulted in the availability of more datasets with longer follow ups (compared to OCT). Second, VFs are already in numeric format and appropriate for most conventional machine learning models which is not the case for fundus images. AI-enabled models for detecting glaucoma progression based on fundus images do exist, but they are rare. This may reflect the fact that quantification of fundus images to provide appropriate input for most conventional machine learning models was challenging. In contrast to fundus imaging, OCT imaging already includes quantification such as retinal thickness profiles that are appropriate for most conventional machine learning models. With the advancement of deep learning models however, more innovative AI models would be desirable in order to fully exploit color fundus photographs as well as raw OCT images for detecting glaucoma progression in clinical practice.

Most current progression detection models utilize statistical approaches based on linear regression, and assume that glaucoma progresses linearly, while there is evidence that glaucomatous progression may be non-linear and rapid, particularly during the later stages.^[119,120]^ Additionally, most of these methods provide only information of whether the eye is progressed or not, without supplemental information on the type of pattern of loss. Therefore, unsupervised machine learning models may offer unbiased analysis of progression and provide explainable outcomes with information on local patterns of loss, rather than a sole binary outcome. As glaucoma is a multifactorial disease caused by a complex interaction of multiple factors, using a comprehensive set of input information may facilitate detection of glaucoma progression. However, no instrument/device yet provides a comprehensive analysis of glaucoma progression based on combined imaging and VF data along with ocular, clinical, and demographic factors. This is an unmet need and future AI models may facilitate detection of glaucoma using multiple sources of information.

### Applications of AI in Estimating Functional Parameters from Structural Factors 

Advancements in AI models in glaucoma have posed critical questions regarding the feasibility of using objective OCT measurements to assess and monitor visual functional loss. A successful solution may replace subjective and tedious VF testing with objective and quick OCT imaging for glaucoma assessment. To that end, numerous teams have attempted to estimate global, regional, and point-wise VF parameters from raw OCT or OCT-derived measurements based on statistical models or machine learning approaches.^[121,122,123]^ Some recent studies have utilized deep learning models to estimate global and local VF damage from raw OCT scans and quantified thickness measurements.^[124,125,126]^ These studies have used scanning laser polarimetry (SLP)-derived RNFL thickness measurements to estimate VF threshold sensitives based on linear and non-linear regression, and obtained approximately 3.9 dB errors (Zhu et al),^[121]^ OCT-derived retinal parameters to estimate VF sensitivities based on support vector regressor machines and achieved a root mean square error (RMSE) of about 3.7 dB,^[122]^ OCT-derived RNFL to estimate VF sensitivities based on deep learning models and obtained RMSE of about 6.1 dB,^[124]^ OCT-derived RNFL thickness measurements to estimate global VF mean deviation (MD) based on deep learning and achieved MAE of about 2.9 dB,^[125]^ raw OCT images from macula and optic disc to estimate VF global parameters based on 3-D deep learning models and obtained RMSE about 2.4 dB and MAE of about 2.3 dB.^[126]^ A follow-up model used an artificial neural network (ANN) model to estimate MD from OCT-derived RNFL parameters based on four independent large datasets from different races, different instruments, and different scanning types and obtained MAE of about 4.0 dB and RMSE of approximately 5.2 dB with reasonable generalizability on other datasets.^[127]^

#### Clinical considerations

Validated AI models may replace VF testing that can be tedious, subjective, and highly variable at later stages of the disease, with OCT imaging that is more objective, quick, and reproducible. Such models could generate quick outcomes and provide more objective glaucoma assessment. However, most of the current models for predicting VF parameters from OCT have several shortcomings. Some of these models underestimate or overestimate global or local VF parameters at both ends of the glaucoma spectrum. Additionally, while the overall error rate of these models may fall within the VF variability, still lower local error rates are required to reach a reasonable level for clinical applications. Finally, the generalizability of these models needs to be evaluated based on representative clinical data to gain clinical utility.

### Unresolved Challenges and Future Directions

As discussed in previous sections, AI models may perform a wide range of tasks such as retinal image and data annotation and interpretation, diagnosis, and prognosis, in order to enhance glaucoma research and clinical practice. Some of the AI models could generate outcomes more quickly, accurately, and consistently than more standard approaches. However, some of the AI models, particularly deep learning, have several limitations as listed below.

Unstable: Deep learning models could be fragile and sometimes even with slightly modifying (unrelated) regions of retina (e.g., by flipping pixels), the diagnosis outcome of the model may change.

Biased: Embedded bias in data could simply become integrated into the model. An AI model that has been trained on retinal images from glaucoma subjects at later stages will be biased and may simply miss patients at early stages of the disease.

Memory: Models may lose their previous ability if retrained on new data. An AI model that was trained on retinal images from glaucoma patients at the early stages of the disease may lose its capability if retrained on new data from subjects in later stages of the disease. The model may simply forget its previous capabilities.

Unexplainable: While there have been efforts in explaining the outcome of deep learning models, still explainablity is a critical challenge in applications in ophthalmology. Clinicians better trust models that explain reasons why they have made a decision rather than providing a sole binary diagnosis.

Uncertain: Except in rare cases,^[128]^ most deep learning models only provide the likelihood of diagnosis and not certainty. In contrary to human expertise that may be wrong on challenging cases, a deep learning model be wrong on simple cases (provide a high likelihood on a definitely wrong decisions).

Foolish: Despite remarkable outcomes in some applications, deep learning models may make foolish mistakes. For instance, a model that has been developed to diagnose glaucoma based on fundus photographs may say a subject has glaucoma from an irrelevant input picture of the lung, while human experts won't make such simple mistakes.

Addressing some of these challenges are the bases of currently active research areas and innovative solutions are becoming increasingly available. In addition to technical challenges, other limitations have hindered widespread clinical utility of AI and deep learning models in glaucoma clinical practice. Some of these challenges are as follows.

Challenges related to glaucoma definitions:

Inconsistent definition of glaucoma: There is not yet a widespread consensus on glaucoma definition and different studies and guidelines have used different definitions.^[129,130]^

Inconsistent definition of glaucoma progression: The problem is even worse for progression as there is no consensus on glaucoma-induced changes as well as a level that constitute a real change.

Challenges related to training and testing AI models:

Most of these reference datasets are annotated by non-ophthalmologists, ophthalmologists, or glaucoma experts with diverse levels of expertise and considerable intra-and inter-rater variability rates. Evaluating AI models based on different datasets thus leads to different levels of performance.

The datasets may be selective and may not represent a diverse group of patients with different ethnicities, required phenotypes, or different severity levels thus generating selective bias.

Datasets are not from the targeted AI model. For instance, the AI model is for proposed for glaucoma screening, but training data is collected from subjects visiting a tertiary eye hospital.

Challenges related to integration of AI in glaucoma clinical practice:

Performance evaluation: Some of the AI model have reported studies that are not based on AI-related study design and reporting guidelines^[131,132,133]^ leading to challenges in validating those models.

Acceptable performance (specificity and sensitivity): The required performance level of a model for glaucoma screening is different from a model targeted for glaucoma diagnosis or prognosis. These levels are not well-defined in glaucoma screening and diagnosis. For instance, as glaucoma is a low- prevalence disease, highly specific and sensitive models are required for screening.

In some AI models, it is confusing whether the model is assistive or autonomous and subsequently whether the AI model has been validated logically. While most of the AI models for retinal image quantification and interpretation are assistive AI tools, most of the diagnostic AI models are autonomous in generating a diagnosis. For instance, if a model is assistive, the evaluation phase requires involvement of the physicians/glaucoma specialists as well. Thus, evaluating the AI model alone limits clinical utility.

Other broad challenges related to integration of AI in glaucoma clinical practice:

Standards for oversight of Software: In different countries, it is still challenging to understand who will oversight an AI system in clinical practice? Developers, physicians, clinics, or providers? These need to be elucidated and each country's regulations may impact this differently.

Liability: It is vital to determine who is (are) responsible for a misdiagnosis or missed diagnosis. These are highly dependent on local regulations and legal systems as well.

Ethical considerations: The minimum requirement for AI models is to not to harm patient. The broader view is that these models would also need to benefit patients and improve clinical and patient outcome.

Reimbursement: Issues need to be resolved for reimbursement and revenue sharing with those who are involved in clinical care, if these AI systems are to receive widespread clinical utility.

Sharing and privacy: The minimum requirement for AI models is that they not violate patient safety and privacy. There are several additional aspects to be clarified, including dataset sharing and who owns the datasets used to train AI models.

##  Summary

AI has shown tremendous potential in both research and clinical treatment of glaucoma. Various conventional AI and emerging deep learning models have been proposed to quantify retinal images and VFs in order to screen, diagnose, forecast, and prognose glaucoma. Some of the AI assistive models have already been integrated in some glaucoma imaging and VF instruments; however, no autonomous AI model has yet received US regulatory approvals to be used in glaucoma care. While there are many challenges regarding integration of AI in glaucoma clinics, a major challenge is the lack of a widely used reference standard for glaucoma, as most of the AI models are trained based on datasets that are subjectively evaluated based on different definitions of glaucoma or its progression. Other challenges include lack of standardized evaluation and reporting of the performance of AI models, targeted patient populations, and liability and ethical issues. Nevertheless, AI applications can provide major improvements in several important areas including *glaucoma research* by setting common grounds for reproducible factors, screening programs with highly specific and sensitive autonomous models for detecting glaucoma, clinical care with establishing assistive and autonomous glaucoma models for delineating hallmarks and diagnosis, and in clinical trial design by identifying subjects and even offering novel digital endpoints.

##  Financial Support and Sponsorship 

This work was supported by the National Institute of Health (NIH) grants EY033005, EY031725, and a Challenge Grant from Research to Prevent Blindness (RPB), New York. The funders had no role in study design, data collection and analysis, decision to publish, or preparation of the manuscript.

##  Conflicts of Interest

The author has no relevant conflict of interest to disclose.
